# Seasonality and Delirium Tremens in Hospitalized Patients with Alcohol Dependence Syndrome

**DOI:** 10.1159/000527973

**Published:** 2023-01-23

**Authors:** Ildikó Katalin Pribék, Bettina Kata Kádár, Lea Péter, Júlia Daróczy, András Bajsz, Csenge Sára Kovács, Ildikó Demeter, Zoltán Janka, Róbert Urbán, Zsolt Demetrovics, Bence András Lázár, Ildikó Kovács, János Kálmán, Bálint Andó

**Affiliations:** ^a^Addiction Research Group, Department of Psychiatry, University of Szeged, Szeged, Hungary; ^b^Department of Psychiatry, University of Szeged, Szeged, Hungary; ^c^Department of Child and Adolescent Psychiatry, University of Szeged, Szeged, Hungary; ^d^Institute of Psychology, ELTE Eötvös Loránd University, Budapest, Hungary; ^e^Centre of Excellence in Responsible Gaming, University of Gibraltar, Gibraltar, Gibraltar

**Keywords:** Delirium tremens, Seasonality, Retrospective examination

## Abstract

**Introduction:**

Due to the high rate of mortality, recognizing the contributing factors of alcohol-related delirium tremens (DT), which is the most severe form of alcohol withdrawal state (AWS) is pivotal in clinical settings. Previous studies suggested relationship between seasonality and other types of delirium; however, to our knowledge, this is the first empirical study which examined the role of seasonality in DT in alcohol dependence syndrome (ADS).

**Methods:**

A retrospective study was undertaken between 2008 and 2015; medical records of 1,591 patients were included, which yielded 2,900 hospital appearances. Three groups were formed based on the ICD-10 diagnoses: ADS, AWS, and DT. The characteristics of the groups were analysed with one-way ANOVA and χ<sup>2</sup> tests. Multinomial logistic regression was used to explore the potential predictors of DT, including seasonality.

**Results:**

The highest incidence of DT was in spring (36.8%; χ<sup>2</sup> (3) = 27.666; *p* < 0.001), especially in March (13.9%; χ<sup>2</sup> (11) = 33.168; *p* < 0.001). Spring, higher mean age, higher presence of comorbid somatic disorders, and lower occurrence of comorbid psychiatric disorders were significant predictive variables for DT with the control of socio-demographic and clinical variables.

**Conclusions:**

The present study revealed that spring, especially March is a critical period in temperate climate zone regarding DT. This can be interpreted as a late winter effect since the temperature is lower in this month compared to other spring months. Furthermore, higher age and the occurrence of comorbid somatic disorders can be considered as risk factors in case of DT. These results support the need of further clinical studies to better understand the impact of seasonality on DT.

## Introduction

Delirium tremens (DT) is the most severe eventuality on the spectrum of alcohol withdrawal state (AWS), which occurs in 5–12% of patients with alcohol dependence syndrome (ADS) [1, 2]. In case of AWS, the symptom characteristics are usually described by tremor, anxiety, paroxysmal sweats, headache, nausea, and insomnia [3, 4]. Additionally, more severe symptoms can appear during the course of DT, such as hallucinations, disorientation, and severe autonomic hyperactivity, which can lead to life-threatening complications [3]. Delirium has severe potential consequences with high rates of morbidity and mortality due to malignant arrhythmia, respiratory arrest, prolonged seizures, and subsequent trauma [5]. Despite the high rates of mortality, delirium is often underdiagnosed due to its fluctuating nature [6] and the high rates of existing comorbid conditions [7]. Therefore, the recognition of possible contributing factors is essential in the prevention of DT.

Several potential socio-demographic and clinical predictors were identified regarding DT such as older age [8, 9], high blood pressure [10] and pulse [11], recent withdrawal seizures, episodes of delirium [10, 11, 12], and the co-occurrence of somatic disorders (e.g., diabetes mellitus, coronary artery disease, hypertension, or chronic obstructive pulmonary disease) [10, 12]. Furthermore, DT also has some sociological risk factors (e.g., employment status or homelessness) [13]; therefore, the role of environmental factors like seasonality should also be examined as potential contributing factors in the incidence of DT.

Hence, the effect of seasonality on the human body has already been investigated from numerous aspects. For instance, it has been demonstrated that temperature and sunshine duration affect vitamin level, biological rhythm and also have an impact on physical activity and mood [14, 15, 16, 17]. The influence of seasonality was also reported in connection with the development of some somatic (e.g., acute pancreatitis or tuberculosis [18, 19]) and psychiatric diseases (e.g., seasonal affective disorder [20]). Additionally, seasonal patterns contribute to various health risk behaviours; for instance, seasons influence the amount and frequency of exercise, smoking status [21], and alcohol consumption [22, 23]. As reported by Cho et al. [22], heavy episodic drinking behaviour was increased during January and July [22]. Furthermore, Carpenter [23] also corroborated this “January effect,” which meant elevated alcohol consumption among participants in January compared to other months. These findings are consistent with the results of Ventura-Cots et al. [24], which showed that colder weather and fewer hours of sunshine may contribute to the higher level of alcohol consumption; therefore, these climate parameters may play a role in alcohol-attributable somatic consequences (e.g., alcohol cirrhosis).

Nevertheless, further complications of excessive alcohol consumption, as well as ADS have been scarcely investigated from the perspective of seasonality. For instance, the association between seasonality and DT is still an unexplored field. However, further types of delirium were examined regarding seasonal patterns. As reported by Balan et al. [25], the incidence of different forms of delirium in geriatric patients was the highest in winter. These findings are in accordance with a study that have shown that delirium in general is more frequent in autumn and winter, especially in January [6].

In conclusion, the importance of seasonality has been suggested in recent empirical research regarding various somatic and mental disorders [18, 19, 20]. Nevertheless, to the best of our knowledge, there is no available data on the association between seasonality and DT, notably on an inpatient sample diagnosed with ADS. However, understanding the relationship between these factors can improve the effectiveness of treatment by taking into account the potential predictors of DT. Thus, the objectives of the present study were (1) to investigate the seasonal and monthly incidence of DT and (2) to explore whether seasonality can be considered as a contributing factor in DT when controlling for general socio-demographic and clinical variables.

## Material and Methods

### Data Collection

A retrospective study was conducted with the aim of collecting medical records of patients with ADS at the Department of Psychiatry, University of Szeged, Hungary between 2008 and 2015. The included 1,591 hospitalized patients had a total of 2,900 appearances during the examined time interval at the unit specialized for the treatment of addiction. Records of patients were eligible for inclusion in the present study in case they had diagnoses related to ADS (F10.2, F10.3, and F10.4) based on the 10th version of the International Classification of Diseases (ICD-10) [26].

Three groups were formed based on the ICD-10 [26] diagnoses of patients. Patients with F10.2 diagnosis who showed the symptoms of ADS, but, did not show withdrawal symptoms were assigned to the (1) ADS group. If symptoms of alcohol withdrawal after the cessation or reduction of alcohol use had been documented, patients were classified into the (2) AWS (F10.3), or the (3) AWS with delirium (DT; F10.4) groups depending on whether the withdrawal was complicated by delirium or not.

Demographic data (sex and age), seasonality (defined by meteorological seasons: winter [1 December–28/29 February], spring [1 March–31 May], summer [1 June–31 August], and autumn [1 September–30 November]), year and month of admission were collected from patients' clinical case histories. Having an address (i.e., not being homeless) as a socio-economic variable was also gathered. In addition, co-occurrence of somatic or psychiatric diseases (besides AWS and DT) was collected in all cases where at least one appearance was registered in patients' history. Based on the severity of their physiological state, every patient received pharmacological treatment (e.g., benzodiazepines and/or antiepileptic drugs).

### Statistical Analysis

IBM SPSS 24 was used for statistical analysis [27]. The unit of data analysis was the total number of hospital appearances during the examined time interval (*N* = 2,900). Firstly, we conducted the analysis of differences between the three groups (ADS, AWS, and DT) regarding socio-demographic variables since these were controlled in the subsequent exploration of potential predictors of AWS and DT. One-way ANOVA was calculated to explore age differences, χ^2^ tests were used to analyse the rate of homelessness and the presence of comorbid somatic and psychiatric disorders. Further χ^2^ tests were also conducted to explore the rates of the three groups regarding seasonality and to examine the monthly and seasonal breakdowns of DT.

Two multinomial logistic regression analyses were conducted to compare the predicting variables for the three groups. In case of the first multinomial logistic regression analysis, the dependent variable was the three-group distinction, with the ADS group as the reference group. In the second multinomial logistic regression, the dependent variable was also the three-group breakdown, but with the AWS group as the reference group. The independent variables were the same factors for both multinomial logistic analyses (age, sex, homelessness, comorbid somatic disorder, comorbid psychiatric disorder, and seasonality). These socio-demographic and clinical variables were also controlled in the analyses. Statistical significance was considered if *p* < 0.05.

## Results

### Sample Characteristics

During the examined 8-year period, 2,900 hospital appearances were registered in the addiction ward (number of patients: 1,591) with relevant ICD-10 diagnoses. In the total sample, 17.3% of the appearances were characterized with ADS without AWS and DT (ADS group; *N* = 502), 70.5% of the appearances developed AWS without DT (AWS group; *N* = 2,045), and 12.2% of the appearances developed DT (DT group; *N* = 353). Socio-demographic variables were compared in Table 1 in case of ADS, AWS, and DT. In the total sample, the mean age at admission was 49.69 (SE = 0.21). ADS, AWS, and DT groups differed significantly in terms of mean age (F(2) = 51.496; *p* < 0.001). Based on the results, the mean age was significantly higher in the DT group (M = 55.21; SE = 0.552) than in the ADS (M = 49.73; SE = 0.576) (*p* < 0.001) and AWS (M = 48.71; SE = 0.238) (*p* < 0.001) groups. In the sample, 1,250 male (78.6%) and 341 female patients (21.4%) were included with no significant differences observed between the ADS, AWS, and DT groups (χ^2^ (2) = 1.317; *p* = 0.518). At the time of treatment, 182 appearances (6.3%) were homeless and the rate of homelessness did not differ significantly between the three groups (χ^2^ (2) = 2.079; *p* = 0.354).

**Fig. 1:**
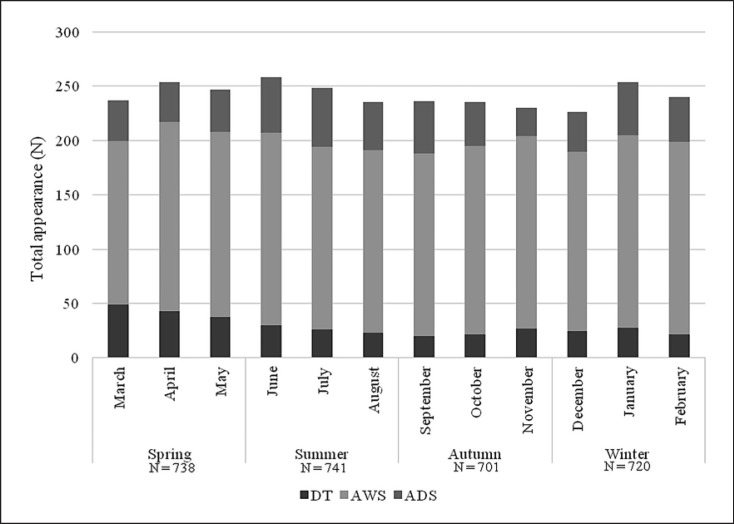
Number of total appearances in the DT, AWS, and ADS groups in regard of seasonality. ADS, alcohol dependence syndrome without alcohol withdrawal state or delirium tremens; AWS, alcohol withdrawal state without delirium tremens; DT, delirium tremens.

A significant association was revealed between DT and seasonality: the highest incidence of DT was in spring (36.8%) compared to other seasons (χ^2^ (3) = 27.666; *p* < 0.001). In regard of months, the highest incidence of DT was in March (*N* = 49; 13.9%) (χ^2^ (11) = 33.168; *p* < 0.001) (shown in Fig. 1).

### Existing Comorbid Somatic and Psychiatric Disorders

The co-occurrences of existing somatic and psychiatric disorders were analysed in the three groups. As detailed in Table 2, the rate of existing comorbid somatic diseases showed a significant difference in the three groups (χ^2^ (2) = 120.847; *p* < 0.001); DT had the highest rate (73.08%) of co-occurring somatic diseases. As for concurrent mental disorders, the three groups also differed significantly (χ^2^ (2) = 178.239; *p* < 0.001) with ADS having the highest rate (68.92%).

### Predictors of AWS and DT

In the first multinomial logistic regression, the potential predictors of AWS and DT were examined by a multinomial logistic regression analysis. In this model, the reference group was the ADS group. Based on the results, younger age (OR = 0.990; 95% CI = 0.981–0.999) and lower co-occurrence of psychiatric disorders (OR = 0.419; 95% CI = 0.339–0.518) were identified as significant predictive factors for the AWS group compared to the ADS group. Regarding the DT group, spring proved to be a significant predictive variable compared to the ADS group (with the season of winter as a reference group) when controlling for general socio-demographic and clinical variables. Based on these results, the chance of DT was almost 2 times higher in spring (OR = 1.928; 95% CI = 1.288–2.883); therefore, spring was considered to be a critical period in terms of the emergence of more severe complications of alcohol withdrawal. Furthermore, higher age (OR = 1.034; 95% CI = 1.020–1.048), elevated presence of comorbid somatic disorders (OR = 2.963; 95% CI = 2.168–4.049) and lower co-occurrence of psychiatric disorders (OR = 0.142; 95% CI = 0.103–0.196) were also significant predictors regarding the DT group.

In the second multinomial logistic regression, the predictors of ADS and DT were also analysed with the reference group being the AWS group. Regarding the ADS group, higher age (OR = 1.010; 95% CI = 1.001–1.019) and higher co-occurrence of psychiatric disorders (OR = 2.386; 95% CI = 1.932–2.947) were significant predictive factors, which were independent from socio-demographic and clinical variables. Considering the DT group, higher age (OR = 1.044; 95% CI = 1.032–1.056), higher presence of comorbid somatic disorders (OR = 2.677; 95% CI = 2.060–3.478) and lower co-occurrence of psychiatric disorders (OR = 0.339; 95% CI = 0.258–0.446) and the season of spring (OR = 1.751; 95% CI = 1.263–2.427) were significant predictive variables compared to the AWS group (with the season of winter as a reference group) when controlling for general socio-demographic and clinical variables. These results also underscore that spring is a notable factor in DT (Table 3).

## Discussion

In the present study, the seasonal and monthly incidence of DT was examined. The purpose of the current investigation was to explore whether seasonality could be presented as a contributing factor in DT in patients diagnosed with ADS when controlling for general socio-demographic and clinical factors. The role of seasonality in connection with psychiatric disorders has already been demonstrated (i.e., seasonal affective disorders). However, based on our literature search, this was the first study evaluating the relationship between seasonality and DT among an inpatient population diagnosed with ADS. Our findings propose that higher age, elevated presence of comorbid somatic disorders and seasonality had a crucial role in the emergence of DT, while lower co-occurrence of other psychiatric disorders among patients diagnosed with alcohol-related mental disorders suggests that patients included in this study have a primary, hence a more severe ADS which might indicate higher incidence of DT. The highest rate of the incidence of DT was in spring, especially in March. These results may contribute to the dissemination of information with regard to the importance of the effect of seasonality in psychiatry, which should be incorporated into the treatment regimen of addiction wards. According to the further socio-demographic analyses, the mean age was the highest in the DT group compared to the ADS and AWS groups.

Up to now, the relationship between the complications of ADS and seasonality has received scarce attention in the scientific literature. Gippini Pérez et al. [28] demonstrated that the most common occurrence of DT was in spring; however, it should be noted that their investigation was conducted at an internal medicine department in Spain where the climate is warmer, and the average temperature in March is also higher compared to Hungary. Previous studies suggested that several types of delirium (e.g., sub-acute delirium, acute delirium, pre-senile dementia with delirium, senile dementia with delirium, or drug-induced delirium) had an elevated occurrence in winter [25]; yet, regarding alcohol-related delirium, our results do not conform to these findings, since the highest incidence of DT in inpatient population diagnosed with ADS was in spring, especially in March. However, it should be taken into account that the increase in the incidence of DT in March may also be due to a late winter effect. In Hungary, this means that the temperature in March is lower compared to other spring months. In conclusion, the low temperature in March is more similar to the temperature in February. These results underscore the importance of further examination concerning the impact of seasonality on DT in patient care from a broad clinical perspective.

The colder weather and decreased hours of sunshine cause potentially clinically significant changes in immunity and vitamin levels [15] which may manifest in increased somatic vulnerability during winter and early spring. Furthermore, patients with ADS, as a consequence of insufficient food intake, frequently have deficiencies in vitamins such as vitamin A, C, D, K, and B and minerals such as magnesium, calcium, iron, or zinc [29, 30, 31, 32, 33, 34]. Consequently, the emergence of these two factors may contribute to the development of DT during winter and early spring due to the aforementioned increased somatic vulnerability.

It has also been proposed that decreased mood, low temperature and fewer hours of sunshine could be potential causal agents for higher incidence of alcohol consumption. For instance, weather highs and lows increase in spring and these atmospheric phenomena with climate change can also affect mood [35], which is related to the elevated frequency of drug and alcohol consumption due to the attempt to alleviate anxiety and stress with them [36, 37]. Moreover, the cold temperature might increase alcohol intoxication through its effects on different oxidative mechanisms [38]. These findings may also serve as a potential explanation of higher incidence of DT in spring.

Regardless the effects of seasonal patterns and weather conditions, it has been documented that the co-occurrence of somatic disorders such as liver disease [39], infections [12], and thrombocytopenia [40] has a pivotal influence on DT. According to our results, the rate of somatic comorbidity was the highest (73.08%) in the DT group. In our study, the following comorbid somatic diagnoses occurred: diseases of the circulatory system, nervous system, digestive system, respiratory system, endocrine, nutritional, and metabolic diseases and injuries, poisoning, and other somatic consequences of external causes. In addition, the crucial role of somatic comorbidity was also emphasized in our study regardless of the effect of general demographic and clinical variables. According to the results, the chance of DT was almost 3 times higher in case of the co-occurrence of somatic disorders. In accordance with the present findings, previous studies have also demonstrated that an existing concurrent somatic state could lead to DT [41, 42]. The association between climate, drinking habits and complications of ADS indicate the importance of the prevention of DT in spring. Further follow-up studies are needed to examine the relationship between different climates and drinking habits, vitamin deficiency, and somatic conditions as well as to investigate the potential role of these in the background of the appearance of DT.

Considering the socio-demographic and further clinical variables of the complications of ADS, the importance of age and the co-occurrence of psychiatric disorders was indicated in the present study. Based on our findings, age was a significant predictor in case of the ADS, AWS, and DT groups. In fact, higher mean age was an essential contributing factor regarding the DT group. This underscores that the highest mean age was shown among the DT group which is consistent with previous literature [9, 43]. Generally, higher age may contribute to the complications of ADS since older patients may be exposed to alcohol consumption for a longer period of time and have a potentially higher comorbidity of other existing somatic or psychiatric conditions [44]. In addition, the co-occurrence of psychiatric diagnoses was also highlighted in the present study since the chance of ADS was more than 2 times higher regarding the presence of psychiatric comorbidity, and it was a significant negative predictor in case of the AWS and DT groups. According to these findings, ADS showed the highest rate regarding comorbid psychiatric disorders and the lowest rate was in the DT group. A possible explanation may be for the highest rate of concomitant psychiatric disorders in the ADS group that DT is the most severe state of the ADS spectrum [4], which is described by numerous acute autonomic and psychiatric symptoms. Therefore, patients may develop a life-threatening condition during DT, which might mask other psychiatric disorders. Consequently, since the primary consideration was to treat this life-threatening condition, it may have concealed other psychiatric disorders during the treatment of DT.

Some limitations of the present study should be taken into account. Firstly, definition of seasons can differ across climate zones, countries, and cultures [45]. Thus, our findings regarding the effect of seasonality in DT can only be interpreted in regions within temperate climate zones. Furthermore, the generalisability of these findings is limited since the data collection was from one hospital in Szeged, Hungary and the associated population living in neighbouring areas. An additional limitation of the study is that the temperature in Szeged was not included in the analyses as a variable, which would have allowed further analysis of the potential association of actual measured temperature and seasonality in connection with the diagnosis of DT. In addition, the clinical profiles of hospital attendances were observed at admittances; therefore, the current state of the patients was emphasized in the present study. In the future, case-control or follow-up studies are vital for a more detailed assessment regarding the contributing factors over time. Furthermore, further studies are needed to explore the intravariability of patients, which provides a great insight into examining the change of seasonal and clinical patterns in case of more hospital admissions. In addition, analysing other parameters (vital signs, laboratory parameters, previous AWS or DT, and predictors of mortality during DT) would also be vital in the future to better understand the contributing factors of DT. Further empirical investigations will need to be undertaken to reveal the impact of seasonality, clinical and alcohol-related variables (e.g., quantity and frequency of drinking) on DT, which may serve as a good basis to prevent the emergence of complications of ADS.

In conclusion, DT has numerous contributing factors. Due to the high rate of mortality, the early recognition of these factors has a crucial role in clinical care. The present study suggests that spring is a critical season in a temperate climate zone concerning DT in an inpatient sample diagnosed with ADS. It also highlights the role of considering other background factors in DT, such as age and comorbid somatic diseases. These results may contribute to the prevention of DT by helping clinicians in recognizing patients who have a potential risk of DT.

## Statement of Ethics

The study was conducted in accordance with the Declaration of Helsinki and was approved by the Human Investigation Review Board, University of Szeged (ethical approval number: 30/2016-SZTE). In this retrospective study, the need for written and informed consent was waived by the Human Investigation Review Board, University of Szeged.

## Conflict of Interest Statement

Zsolt Demetrovics declares that he is Editorial Board Member of the European Addiction Research.

## Funding Sources

This work was supported by the Hetényi Géza Grant (SZTE-ÁOK-KKA-2019-HG). Zsolt Demetrovics's and Róbert Urbán's contribution was supported by the Hungarian National Research, Development and Innovation Office (KKP126835; ELTE Thematic Excellence Programme 2020, KP2020-IKA-05, K131635; FK134807). Ildikó Katalin Pribék and Lea Péter were supported by the University of Szeged, Faculty of Medicine (EFOP-3.6.3-VEKOP-16-201700009). Bence András Lázár was supported by the ÚNKP-22-306 New National Excellence Program of the Ministry for Culture and Innovation from the source of the National Research, Development and Innovation Fund.

## Author Contributions

András Bajsz, Bálint Andó, Ildikó Kovács, János Kálmán, Bence András Lázár, and Zoltán Janka designed and conceptualized the study. Ildikó Katalin Pribék and Lea Péter conducted literature search. Ildikó Kovács, Júlia Daróczy, Bálint Andó, Csenge Sára Kovács, Ildikó Demeter, and Ildikó Katalin Pribék contributed to data collection, organization, and curation. Ildikó Katalin Pribék, Zsolt Demetrovics, and Róbert Urbán conducted the data analysis. Ildikó Katalin Pribék, Bettina Kata Kádár, Lea Péter, Júlia Daróczy, András Bajsz, Csenge Sára Kovács, Ildikó Demeter, Zoltán Janka, Róbert Urbán, Zsolt Demetrovics, Bence András Lázár, Ildikó Kovács, János Kálmán, and Bálint Andó contributed to the data interpretation, and Ildikó Katalin Pribék, Lea Péter, and Bálint Andó wrote the first version of the manuscript. Ildikó Katalin Pribék, Bettina Kata Kádár, Lea Péter, Júlia Daróczy, András Bajsz, Csenge Sára Kovács, Ildikó Demeter, Zoltán Janka, Róbert Urbán, Zsolt Demetrovics, Bence András Lázár, Ildikó Kovács, János Kálmán, and Bálint Andó contributed to providing edits and feedback to manuscript drafts and contributed and approved the final version of the manuscript.

## Data Availability Statement

The dataset of the study is available from the corresponding author (Bálint Andó) upon request. This anonymised dataset has been generated from the registered official health insurance patient flow of the university clinic, and due to the official data protection policy, these data are preferred not to be made fully available but only on request. Further enquiries can be directed to the corresponding author (Bálint Andó).

## Figures and Tables

**Table 1:** Characteristics of hospital appearances in regard of ADS and its complications (AWS or DT)

	ADS (*N* = 502)	AWS (*N* = 2,045) DT (*N* = 353)	Sig. (*p* value)
Mean age (SE)	49.73 (0.576)	48.71 (0.238) 55.21 (0.552)	<0.001
Male sex, *N* (%)	386 (76.89)	1,619 (79.17) 280 (79.32)	0.518
Homelessness, *N* (%)	26 (5.19)	137 (6.7) 19 (5.41)	0.354
Seasons, *N* (%)			<0.001
Spring	113 (22.5)	495 (24.2) 130 (36.8)	
Summer	149 (29.7)	513 (25.1) 79 (22.4)	
Autumn	114 (22.7)	518 (25.3) 69 (19.5)	
Winter	126 (25.1)	519 (25.4) 75 (21.2)	

ADS, alcohol dependence syndrome without alcohol withdrawal state or delirium tremens; AWS, alcohol withdrawal state without delirium tremens; DT, alcohol withdrawal state with delirium; SE, standard error; Sig., significance.

**Table 2:** Comorbid somatic and psychiatric disorders recorded in the hospital appearances of patients with ADS and its complications (AWS or DT)

	ADS (*N* = 502)	AWS (*N* = 2,045)	DT (*N* = 353)	Sig. (*p* value)
Comorbid somatic disorders, *N* (%)	198 (39.44)	877 (42.89)	258 (73.08)	<0.001
Comorbid psychiatric disorders, *N* (%)	346 (68.92)	984 (48.14)	80 (22.66)	<0.001

ADS, alcohol dependence syndrome without alcohol withdrawal state or delirium tremens; AWS, alcohol withdrawal state without delirium tremens; DT, alcohol withdrawal state with delirium; SE, standard error; Sig., significance.

**Table 3:** Multinomial logistic regression of the AWS and DT groups versus the ADS group as the reference group

	Model 1			Model 2		
	B (SE)	OR	95% CI	B (SE)	OR	95% CI
	AWS (without DT)			ADS (without AWS or DT)		
Age	−0.010 (0.005)	0.990*	**0.981–0.999**	0.010 (0.005)	1.010*	**1.001–1.019**
Sex: Males (Ref: Females)	0.073 (0.121)	1.076	0.848–1.365	−0.073 (0.121)	0.929	0.733–1.179
Homelessness	0.023 (0.225)	1.023	0.658–1.591	−0.023 (0.225)	0.978	0.629–1.521
Comorbid somatic disorder	0.102 (0.106)	1.107	0.899–1.363	−0.102 (0.106)	0.903	0.734–1.112
Comorbid psychiatric disorder Seasons	−0.870 (0.108)	0.419***	**0.339–0.518**	0.870 (0.108)	2.386***	**1.932–2.947**
Spring	0.096 (0.146)	1.101	0.827–1.466	−0.096 (0.146)	0.908	0.682–1.209
Summer	−0.139 (0.138)	0.870	0.663–1.141	0.139	1.150	0.876–1.508
Autumn	0.150 (0.146)	1.161	0.873–1.545	−0.150 (0.146)	0.861	0.647–1.146
Winter		Ref.			Ref.	

*DT*						
Age	0.033 (0.007)	1.034*	**1.020–1.048**	0.043 (0.006)	1.044*	**1.032–1.056**
Sex: Males (Ref: Females)	−0.073 (0.180)	0.929	0.653–1.322	−0.147 (0.151)	0.864	0.643–1.161
Homelessness	−0.210 (0.325)	0.811	0.429–1.533	−0.232 (0.263)	0.793	0.474–1.326
Comorbid somatic disorder	1.086 (0.159)	2 963***	**2.168–4.049**	0.985 (0.134)	2.677***	**2.060–3.478**
Comorbid psychiatric disorder Seasons	−1.950 (0.164)	0.142***	**0.103–0.196**	−1.081 (0.140)	0.339***	**0.258–0.446**
Spring	0.656 (0.205)	1.928**	**1.288–2.883**	0.560 (0.167)	1.751 ***	**1.263–2.427**
Summer	−0.063 (0.212)	0.939	0.620–1.423	0.077 (0.180)	1.080	0.758–1.538
Autumn	0.110 (0.222)	1.116	0.723–1.724	−0.040 (0.186)	0.961	0.668–1.384
Winter		Ref.			Ref.	

The reference group of Model 1 is alcohol dependence syndrome (without alcohol withdrawal state or delirium tremens). The reference group of Model 2 is alcohol withdrawal state (without delirium tremens). ADS, alcohol dependence syndrome without alcohol withdrawal state or delirium tremens; AWS, alcohol withdrawal state without delirium tremens; DT, delirium tremens; B, unstandardized regression coefficient; CI, confidence interval; OR, odds ratio; SE, standard error; Ref., reference group.

* (*p* < 0.05;

** *p* < 0.01;

*** *p* < 0.001).
